# Accelerated Wound Healing and Keratinocyte Proliferation through PI3K/Akt/pS6 and VEGFR2 Signaling by Topical Use of Pleural Fluid

**DOI:** 10.3390/cells11050817

**Published:** 2022-02-26

**Authors:** Chen-Liang Tsai, Chih-Ying Changchien, Ying Chen, Hsin-Han Chang, Wen-Chiuan Tsai, Yi-Wen Wang, Kai-Chieh Chou, Ming-Hsien Chiang, Yu-Ling Tsai, Hao-Chung Tsai, Chieh-Yung Wang, Ming-Sheng Shen, Li-Ting Cheng, Hung-Yi Lin, Tse-Bin Yang, Chih-Feng Chian

**Affiliations:** 1 Department of Internal Medicine, Division of Pulmonary and Critical Care Medicine, Tri-Service General Hospital, National Defense Medical Center, Taipei 114, Taiwan; doc10376@gmail.com (C.-L.T.); marechaparis@gmail.com (C.-Y.W.); letim47@gmail.com (L.-T.C.); ssbn621@gmail.com (H.-Y.L.); 2 Department of Internal Medicine, Tri-Service General Hospital, National Defense Medical Center, Taipei 114, Taiwan; koala8072@yahoo.com.tw; 3 Department of Biology and Anatomy, National Defense Medical Center, Taipei 114, Taiwan; eva.flower@gmail.com (Y.C.); albertchang1008@gmail.com (H.-H.C.); christmas1035@mail.ndmctsgh.edu.tw (Y.-W.W.); jeffrey8464@gapps.ndmctsgh.edu.tw (K.-C.C.); ph870101@mail.ndmctsgh.edu.tw (M.-H.C.); 4 Department of Pathology, Tri-Service General Hospital, National Defense Medical Center, Taipei 114, Taiwan; ab95057@hotmail.com (W.-C.T.); c909228@gmail.com (Y.-L.T.); 5 Department of Internal Medicine, Division of Chest Medicine, Tri-Service General Hospital Songshan Branch, Taipei 105, Taiwan; petstsai@yahoo.com.tw; 6 Department of Internal Medicine, Taichung Armed Force General Hospital, Taichung 411, Taiwan; darkevilalien@gmail.com; 7 Department of Internal Medicine, Taipei City Hospital Renai Branch, Taipei 106, Taiwan; nothingbut13@gmail.com

**Keywords:** pleural fluid, wound healing, keratinocyte, VEGFR2, pS6

## Abstract

Impaired wound healing is an ongoing issue that cancer patients undergoing chemotherapy or radiotherapy face. Our previous study regarding lung-cancer-associated pleural fluid (LCPF) demonstrated its propensity to promote endothelial proliferation, migration, and angiogenesis, which are crucial features during cutaneous wound healing. Therefore, the current study aimed to investigate the effect of pleural fluid on cutaneous wound closure in vitro and in vivo using HaCaT keratinocytes and a full-thickness skin wound model, respectively. Both heart-failure-associated pleural fluid (HFPF) and LCPF were sequentially centrifuged and filtered to obtain a cell-free status. Treatment with HFPF and LCPF homogeneously induced HaCaT proliferation with cell cycle progression, migration, and MMP2 upregulation. Western blotting revealed increased PI3K/Akt phosphorylation and VEGFR2/VEGFA expression in HaCaT cells. When treated with the PI3K inhibitor, LCPF-induced keratinocyte proliferation was attenuated with decreased pS6 levels. By applying the VEGFR2 inhibitor, LCPF-induced keratinocyte proliferation was ameliorated by pS6 and MMP2 downregulation. The effect of LCPF-induced cell junction rearrangement was disrupted by co-treatment with a VEGFR2 inhibitor. Compared with a 0.9% saline dressing, LCPF significantly accelerated wound closure and re-epithelization when used as a dressing material in a full-thickness wound model. Histological analysis revealed increased neo-epidermis thickness and dermis collagen synthesis in the LCPF-treated group. Furthermore, LCPF treatment activated basal keratinocytes at the wound edge with the upregulation of Ki-67, VEGFA, and MMP2. Our preliminaries provided the benefit of wet dressing with pleural fluid to improve cutaneous wound closure through enhanced re-epithelization and disclosed future autologous application in cancer wound treatment.

## 1. Introduction

Multiple growth factors are involved in cutaneous wound healing through the promotion of the proliferation and migration of resident cells [1]. The process of tissue repair consists of re-epithelization via keratinocytes, the generation of connective tissue via fibroblasts, and neo-vessel formation via endothelial cells [2,3]. In our previous study, cell-free malignant pleural effusion (MPE) significantly stimulated endothelial cells with increased proliferation, migration, and angiogenesis [4,5]. The upregulation of VEGFR2 and VEGFA protein expression has been observed in MPE-cultured endothelial cells [4]. In particular, cell-free MPE did not induce the malignant transformation of endothelial cells [6]. The importance of endothelial cells in wound healing has been well characterized to provide an optimal vascular network [7]. The current study aimed to investigate the potential of pleural fluid in cutaneous wound healing, particularly keratinocytes, based on the positive results of cell-free MPE on the vascular endothelium.

The pleura space contains a small amount of pleural fluid as a lubricant between the visceral and parietal pleura [8]. Massive pleural fluid develops and accumulates in the pleura cavity in patients with heart failure or underlying malignancy [9,10]. To relieve dyspnea symptoms, thoracocentesis can be performed to aspirate pleural effusion (volume > 0.5 L), and patients frequently require repeated thoracocentesis [11]. Massive pleural fluid has previously been shown to provide diagnostic value in differentiating underlying disease, whereas additional values and the possible application of pleural fluid have not been investigated in previous studies.

Deficiencies in growth factors, such as bFGF, PDGF, EGF, VEGF, and TGF-β, have been found to exacerbate delayed wound healing [12,13]. The exogenous application of growth factors has been widely studied as an adjuvant treatment for wound closure [14]. The advantages of growth factor treatment include that it is biosafe and does not cause significant toxicity or adverse reactions [15]. Combinatory growth factors have been proposed for use in the treatment of chronic diabetic wounds [16]. One notable example is the application of autologous platelet-rich plasma (PRP) in various complicated wounds. However, the expansion and preparation of PRP is considerable. Previous analysis of pleural fluid revealed the diversity of cytokines and growth factors [17,18]. Therefore, the current study aimed to investigate the possibility of pleural-fluid-based application in cutaneous wound healing.

Keratinocyte re-epithelization is crucial to the cutaneous wound-healing process, which depends on proper proliferation and migration [19]. Matrix metalloproteinases (MMPs) degrade the extracellular matrix (ECM) and thus regulate the ability of keratinocytes to detach from the basal membrane and migrate [20]. Moreover, the phosphoinositide 3-kinase (PI3K)/Akt pathway plays a critical role in keratinocyte proliferation and differentiation [21]. Targeting PI3K/Akt and mTORc signaling has been shown to restore skin homeostasis and accelerate tissue regeneration in mouse wound models [22,23]. Currently, there is no literature regarding the changes in pleural-fluid-induced keratinocyte signaling. Accordingly, the present study aimed to investigate the biological effects of pleural fluid on keratinocyte proliferation and migration and the application of pleural fluid in cutaneous wound injury.

## 2. Materials and Methods

### 2.1. Patient Characteristics and Collection of Pleural Fluid Samples

The study was approved by the Institutional Review Board of the Tri-Service General Hospital Research Ethics Committee. Under sonography-guided thoracentesis, pleural fluid samples were obtained from heart failure, lung cancer, and breast cancer patients who had provided written informed consent. From each patient, the drained amount of pleural fluid was often more than 500 mL, and we collected, in total, 5 mL of pleural fluid for use in the in vitro and in vivo experiments that followed. Fresh samples were immediately centrifuged at 1000× *g* for 15 min and filtered (0.22 µm; Millipore, Burlington, MA, USA) to obtain a cell-free specimen. All samples were stored at −80 °C and thawed once before use.

### 2.2. Culture of Keratinocytes

HaCaT keratinocytes were purchased from the Bioresource Collection and Research Center (Hsinchu, Taiwan) and cultured in Dulbecco’s modified Eagle medium (ScienCell Research Laboratories, Carlsbad, CA, USA).

### 2.3. Drugs and Reagents

Coomassie brilliant blue G-250 and 3-(4,5-dimethylthiazol-2-yl)-2,5-diphenyltetrazolium bromide (MTT) were purchased from Sigma-Aldrich (St. Louis, MO, USA). Sunitinib, marketed as Sutent, was purchased from Sigma-Aldrich. Bevacizumab, marketed as Avastin, was obtained from Roche (Basel, Switzerland).

### 2.4. Cell Survival Assay

HaCaT cells were plated at a density of 2 × 10^4^ cells per well in a 96-well plate. Pleural fluid was then added to the culture medium with 30% MAPF (*v*/*v*) and incubated for 24 h. After washing the cells with phosphate-buffered saline (PBS), MTT (0.5 mg/mL) was added, and the plates were incubated for another 4 h. Cells were then lysed with DMSO. The absorbance of each well was measured at a wavelength of 590 nm.

### 2.5. Flow Cytometric Analysis

Cell cycle analysis was performed by seeding 2 × 10^5^ HaCaT cells in 6-well plates. After cell attachment, growth medium with or without pleural fluid was added for 24 h. Cells were fixed in ethanol and stained with propidium iodide (PI) for cell cycle assays. The cells were washed with binding buffer (4-(2-hydroxyethyl)-1-piperazineethanesulfonic acid, 140 mmol/L NaCl, and 5 mmol/L CaCl2 at pH 7.4), stained with anti-annexin V antibody (FITC), and then counterstained with PI for 15 min at room temperature. The results were measured using a FACS Verse laser flow cytometric analysis system (Becton Dickinson, Franklin Lakes, NJ, USA). In total, 10,000 cells were analyzed for each sample.

### 2.6. Migration and Transwell Assays

The migration ability of HaCaT cells was assayed using wound-healing and Transwell assays. The wound area was prepared by seeding HaCaT in a 3.5 cm culture dish to form a monolayer. After being scratched with a P200 pipette tip and being photographed, the cells were cultured with 30% pleural (*v*/*v*) for 18 h. The wound area was analyzed using ImageJ software. For the Transwell migration assay, 2 × 10^4^ HUVECs were seeded into the upper chamber of a Transwell plate (Corning Costar, Cambridge, MA, USA). Following incubation at 37 °C for 18 h, the cells on the lower side of the insert were fixed with 10% formalin in PBS and stained with Coomassie Brilliant Blue G250 (Sigma-Aldrich). Migrated cells were examined in three randomly selected fields from each membrane in five independent experiments. 

### 2.7. Western Blotting

HaCaT cells were homogenized using a protein extraction buffer (GE Healthcare Life Sciences, Chicago, IL, USA) with proteinase and phosphatase inhibitors (MedChemExpress, Monmouth Junction, NJ, USA). Electrophoresis was performed on a 10% sodium dodecyl sulfate–polyacrylamide gel electrophoresis gel, and the protein samples were transferred to a nitrocellulose membrane (Bio-Rad, Hercules, CA, USA). Strips from the membrane were incubated with 5% non-fat milk in Tris-buffered saline (pH 7.4) containing 0.1% Tween. Next, the membranes were incubated in blocking solution with primary antibodies overnight at 4 °C. After being washed, the strips were incubated with a 1:5000 or 1:10,000 dilution of horseradish peroxidase-conjugated anti-rabbit or anti-mouse immunoglobulin G (IgG) antibodies from Cell Signaling Technology (Danvers, MA, USA). Subsequently, the blots were incubated in developing solution with an electrochemiluminescence substrate (Bio-Rad). The band densities on the membrane were captured and quantified using ImageJ software. The density of the control sample was set to 100%, and the densities of the test samples were relative to those of the internal control. At least six independent experiments were conducted.

### 2.8. Immunofluorescence Staining

HaCaT cells were seeded on coverslips and incubated in the presence of pleural fluid for 24 h. The cells were then rinsed with PBS and fixed with 10% (*v*/*v*) formalin in PBS (pH 7.4). A blocking solution (5% milk in 0.1% (*v*/*v*) Triton X-100) was applied to prevent nonspecific binding. Primary antibodies against ZO-1 and F-actin in blocking buffer were incubated with HaCaT cells at 4 °C overnight. After the antibody was washed, the slides were incubated with fluorescein isothiocyanate-conjugated goat anti-mouse and anti-rabbit IgG (Sigma-Aldrich) for 1 h. Finally, coverslips were mounted with Gel Mount Aqueous mounting medium (Sigma-Aldrich) and photographed with a Nikon D1X digital camera (Carl Zeiss, Oberkochen, Germany).

### 2.9. Mouse Full-Thickness Wound Model and Daily Change of Wound Dressings

Mice were anesthetized subcutaneously prior to wounding. The dorsal surface was shaved and draped prior to surgery. All wounding procedures and post-operative treatments were performed by the same surgeon. Two full-thickness wounds were created using a 6 mm sterile skin biopsy punch and scissors on one mouse. The wet dressing was replaced daily for three days after the operation. The mice were briefly anesthetized before changing the dressing. Wound beds were photographed on days 0, 1, 2, 3, and 4 using a digital camera with standardized exposure and focal lengths. After sacrifice, the wound skin tissues were dissected and fixed with 10% formalin, embedded in paraffin, and sectioned.

### 2.10. Hematoxylin and Eosin (HE) Staining, Masson’s Trichrome Staining, and Immunohistochemistry

Wounded skin tissues were fixed in 10% (*v*/*v*) formalin, embedded in paraffin, and sectioned at 6 μm using a microtome. The paraffin sections were deparaffinized and stained with HE in a standard manner to assess general tissue morphology. The organization and maturation of collagen bundles was assessed by Masson trichrome. The expression levels of Ki-67, VEGFA, and MMP2 were detected using immunohistochemical staining, which was conducted using the Ventana BenchMark ULTRA system (Roche). The primary antibody was diluted in antibody dilution buffer (Ventana). Antigen retrieval was performed according to the manufacturer’s protocol. Secondary goat anti-rabbit antibodies (Jackson ImmunoResearch Laboratories, West Grove, PA, USA) were used. Protein expression was observed in 6 random fields in each group.

### 2.11. Statistical Analysis

Data are expressed as the average of at least triplicate samples and are presented as the mean ± standard error of the mean. Analysis was performed using Student’s *t*-test, with statistical significance set at *p* < 0.05.

## 3. Results

### 3.1. Effect of Cell-Free Pleural Fluid on Keratinocyte Cell Viability, Motility, and Cell Cycle Progression

**Figure 1:**
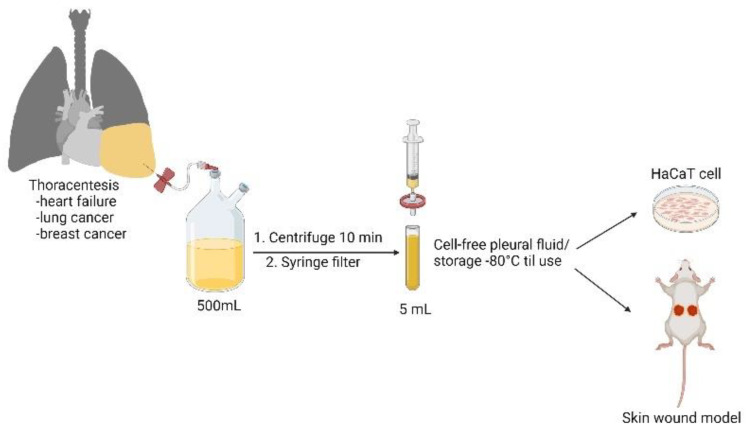
Preparation protocol of cell-free pleural fluid.

**Figure 2:**
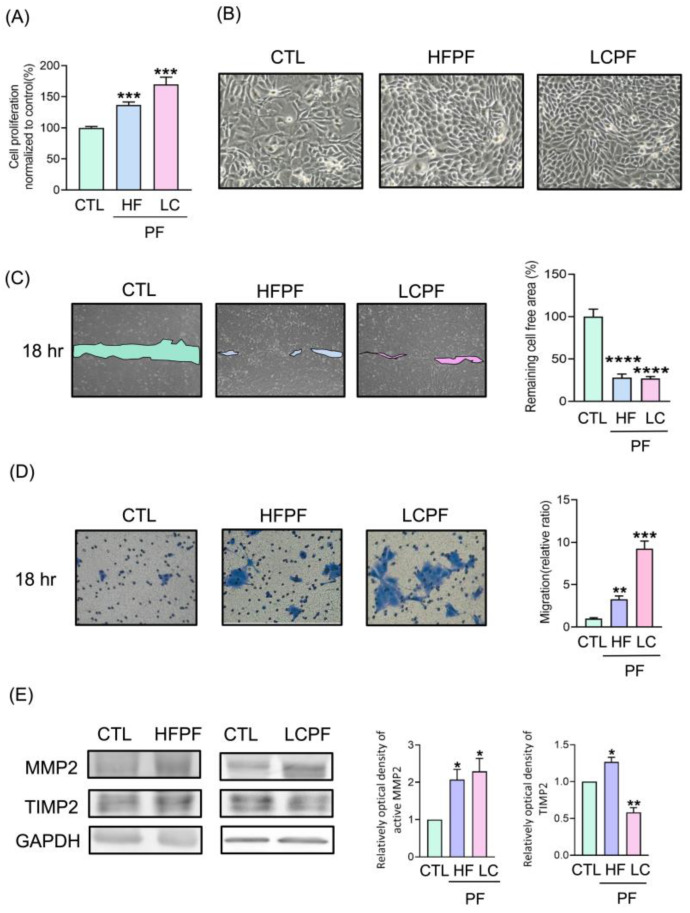
Effect of cell-free pleural fluid (PF) from heart failure (HF) and lung cancer (LC) patients on HaCaT viability and motility. Through sonography-guided thoracentesis, PF from heart failure (HFPF) and lung cancer (LCPF) patients were collected. (**A**) HaCaT cells were cultured with HFPF, LCPF, or control medium for 24 h. Cell viability was determined using an MTT assay. Quantification shown as bar graphs. (**B**) Representative images of HaCaT cells after being cocultured with HFPF and LCPF for 12 h. (**C**) After cells reached confluency, a scratch wound assay was applied. HaCaT cells were treated with HFPF, LCPF, or control medium for 18 h. The wound closure is demonstrated by the colored area. Bar graphs show the quantification of the cell-free area. (**D**) HaCaT was seeded in the upper Transwell chamber. After an 18 h incubation with HFPF or LCPF, the number of cells in the lower chamber was stained and counted. Bar graphs show the quantification of the migration rate. (**E**) Levels of MMP2 and TIMP2 following treatment with HFPF or LCPF for 24 h were measured by Western blotting. GAPDH was used as internal control. * *p* < 0.05; ** *p* < 0.01; *** *p* < 0.005; **** *p* < 0.0001 compared to the control group.

**Figure 3:**
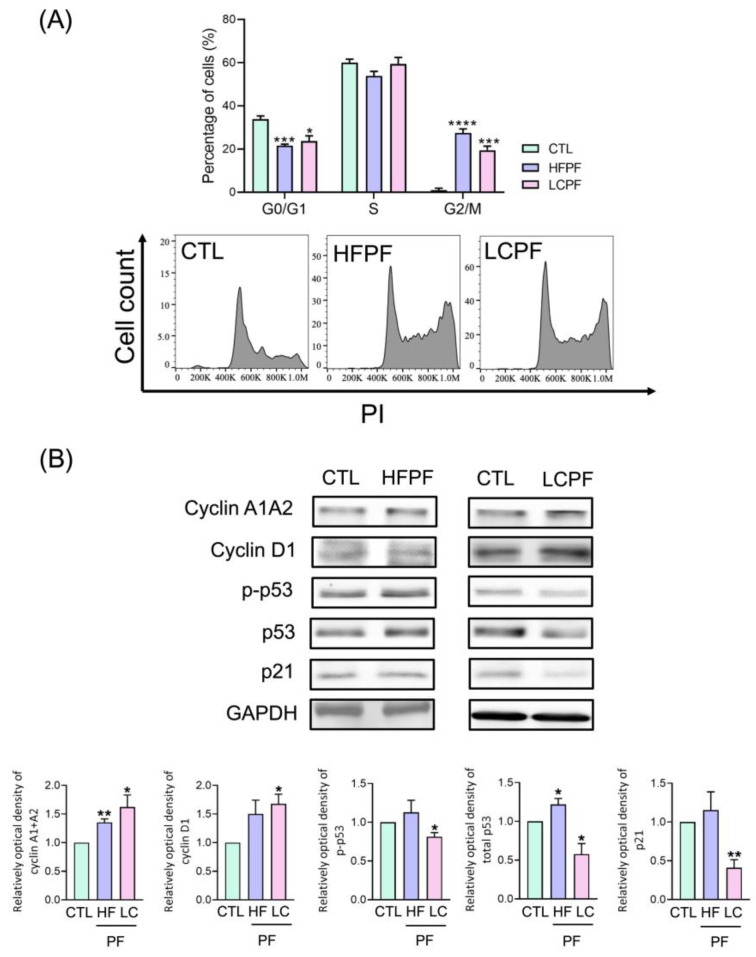
Effect of cell-free HFPF and LCPF on keratinocyte cell cycle regulation. HaCaT cells were treated with HFPF, LCPF, or the control medium for 24 h. (**A**) Flow cytometry was then performed to analyze cell cycle expression. Quantification of cell cycle populations in the G0/G1, S, and G2/M phases was analyzed using BD FACSuite software. (**B**) Protein expression of cyclin A1A2, cyclin D1, *p*-p53, p53, p21, and GAPDH in HaCaT cells. The lower panels show the quantitative analyses of Western blotting. * *p* < 0.05; ** *p* < 0.01; *** *p* < 0.005; **** *p* < 0.0001 compared to the control group.

HaCaT cells were used as an in vitro model to investigate the wound-healing potential of the cell-free pleural fluid. The preparation of cell-free pleural fluid is summarized in Figure 1. As shown in the MTT assay, both HFPF and LCPF treatment revealed a more than 50% increase in HaCaT cell viability at 24 h (Figure 2A). Breast-cancer-associated pleural fluid (BCPF) was also examined with regard to keratinocyte viability. A similar phenomenon was observed in HaCaT cells cultured with BCPF (Figure S1A). At 18 h post-seeding, the morphology of HaCaT cells cultured with HFPF and LCPF presented a more uniformly small size and high nucleus to cytoplasm ratio (Figure 2B). Epithelial cell migration is a crucial step in cutaneous wound healing. From scratch wound and Transwell assays, increased cell motility was observed in HaCaT cells cultured with HFPF or LCPF for 18 h (Figure 2C,D). Culturing with BCPF also showed enhanced keratinocyte migration (Figure S1B). MMP-2 (MMP2) and tissue inhibitor of metalloproteinase-2 (TIMP2) regulated ECM degradation and deposition for re-epithelization steps during wound healing [24]. Western blotting showed that HFPF and LCPF treatment significantly upregulated MMP2 expression in HaCaT cells in 24 h (Figure 2E). Compared with that of the control group, LCPF downregulated TIMP2 expression in keratinocytes, whereas increased TIMP2 expression was observed in the HFPF group. HaCaT cells cultured with BCPF showed similar trends in MMP2 and TIMP2 protein levels in the LCPF group (Figure S1C). Cell cycle regulation plays a critical role in keratinocyte proliferation during wound healing [24]. After being cultured with HFPF or LCPF for 24 h, HaCaT cells were subjected to flow cytometry for cell cycle analysis. There was an increased percentage of the G2/M phase and a decreased G0/G1 subpopulation in HaCaT cells cultured with HFPF or LCPF (Figure 3A). A similar trend of cell cycle analysis was observed in HaCaT cells cultured with BCPF (Figure S1D). For cell-cycle-regulated proteins, LCPF induced cyclin A1A2 and cyclin D1 protein expression (Figure 3B). Moreover, p53 phosphorylation and p21 were downregulated in HaCaT cells cultured with LCPF. Our data revealed the potency of HFPF and LCPF in regulating keratinocyte cell fate through cell cycle progression. The above results demonstrated the ability of HFPF, LCPF, and BCPF to stimulate keratinocyte proliferation, cell cycle regulation, migration, and MMP2 expression. Due to a similar effect on keratinocytes via HFPF, LCPF, or BCPF, the following experiments focused on the HFPF and LCPF groups.

### 3.2. HFPF and LCPF Upregulates p-PI3K/Akt and VEGFR2 Signaling in Keratinocytes

**Figure 4:**
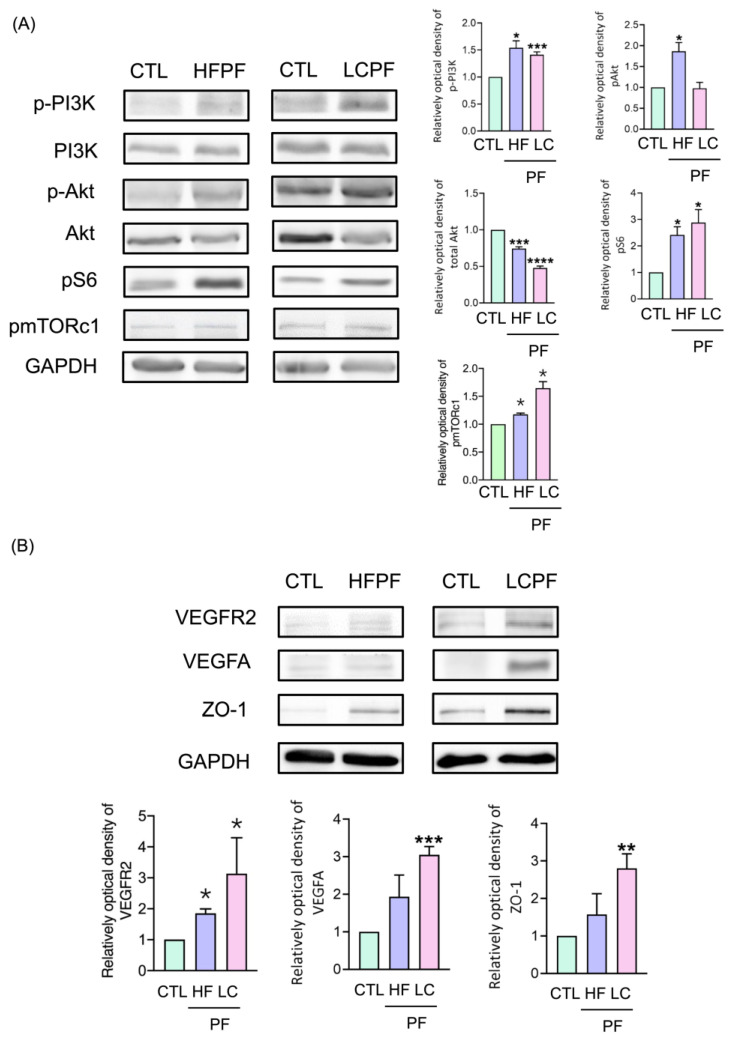
Upregulated PI3K/Akt and angiogenesis signaling via HFPF and LCPF in HaCaT cells. HaCaT cells were individually treated with HFPF, LCPF, or control medium for 24 h. (**A**) *p*-PI3K, *p*-Akt, pS6, and PPARγ, and (**B**) VEGFR2, VEGFA, and Zonula occludens-1 protein expression was examined via Western blot analysis. GAPDH was used as an internal control. * *p* < 0.05; ** *p* < 0.01; *** *p* < 0.005; **** *p* < 0.0001 compared to the control group.

Following increased cell viability, motility, and cell cycle progression in HaCaT cells cultured with HFPF and LCPF, we further examined changes in cell signaling related to wound healing. Increased PI3K phosphorylation was observed in HaCaT cells cultured with HFPF and LCPF for 24 h (Figure 4A). HFPF induced *p*-Akt upregulation in HaCaT cells; however, LCPF did not. Nevertheless, the total Akt expression was downregulated in HaCaT cells cultured with LCPF. mTORC1 and its direct target, S6 kinase, were activated via PI3K and Akt phosphorylation, which regulated keratinocyte proliferation. HaCaT cells cultured with HFPF or LCPF for 24 h showed increased mTORC1 and S6 phosphorylation (Figure 4A). The upregulation of PI3K/Akt signaling and downstream targets mTORC1 and pS6 was observed in keratinocytes, which was compatible with increased cell viability via HFPF and LCPF treatment.

Angiogenesis signaling accelerates cutaneous wound repair [25]. Our previous results showed that LCPF treatment has the capacity to upregulate VEGFR2/VEGFA expression but not ZO-1 expression in endothelial cells [22]. The current study revealed that HFPF or LCPF treatment increased VEGFR2/VEGFA expression in HaCaT cells in 24 h (Figure 4B). ZO-1, a tight junction protein, played a role in angiogenesis and also maintained the epithelial barrier [26]. Elevated ZO-1 expression was observed in HaCaT cells following LCPF culture for 24 h. The current results showed that LCPF and HFPF upregulated VEGFR2/VEGFA expression in HaCaT cells, which could contribute to wound healing. Coculturing with HFPF or LCPF yielded similar phenotypes in HaCaT cells; therefore, LCPF could be applied in the following experiments.

### 3.3. VEGFR2 Inhibitor, But Not p-PI3K Inhibitor, Attenuates LCPF-Induced Cell Viability and MMP2 Expression

**Figure 5:**
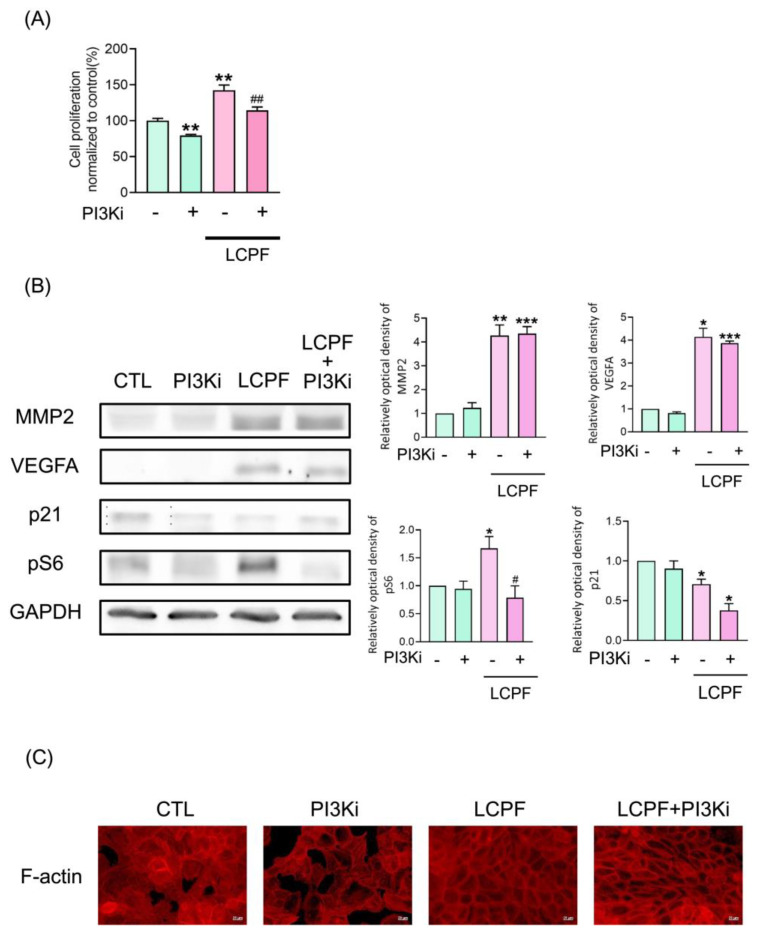
*p*-PI3K inhibitor restores LCPF-induced keratinocyte proliferation but not MMP2, VEGFA expression, and epidermal junction rearrangement. HaCaT was treated with or without 10 µM of *p*-PI3K inhibitor, LY294002, in the presence of LCPF for 24 h. (**A**) Cell viability was determined using an MTT assay. (**B**) MMP2, VEGFA, p21, and pS6 protein expression was examined via Western blot analysis. (**C**) The cells were then subjected to F-actin staining (red). Scale bar = 40 µm. * *p* < 0.05; ** *p* < 0.01; *** *p* < 0.005 compared to the control group. ^#^ *p* < 0.05; ^##^ *p* < 0.01 compared to the LCPF group.

To elucidate LCPF-induced cell signaling changes in HaCaT cells, specific p-PI3K and VEGFR2 inhibitors were used. In the MTT assay, co-treatment with 10 μM of LY294002 as a p-PI3K inhibitor for 24 h significantly suppressed LCPF-induced keratinocyte viability (Figure 5A). A similar effect was observed in HFPF-treated HaCaT cells (Figure S2A). As previously reported [27], the p-PI3K inhibitor downregulated S6 phosphorylation in HaCaT cells (Figure 5B). In contrast, there was negligible change in MMP2, VEGFA, and p21 protein expression in HaCaT cells after co-treatment with LCPF and p-PI3K inhibitors. The above results indicated that the activation of PI3K and pS6 contributed to LCPF-induced keratinocyte proliferation but not LCPF-regulated MMP2 and VEGFA expression.

**Figure 6:**
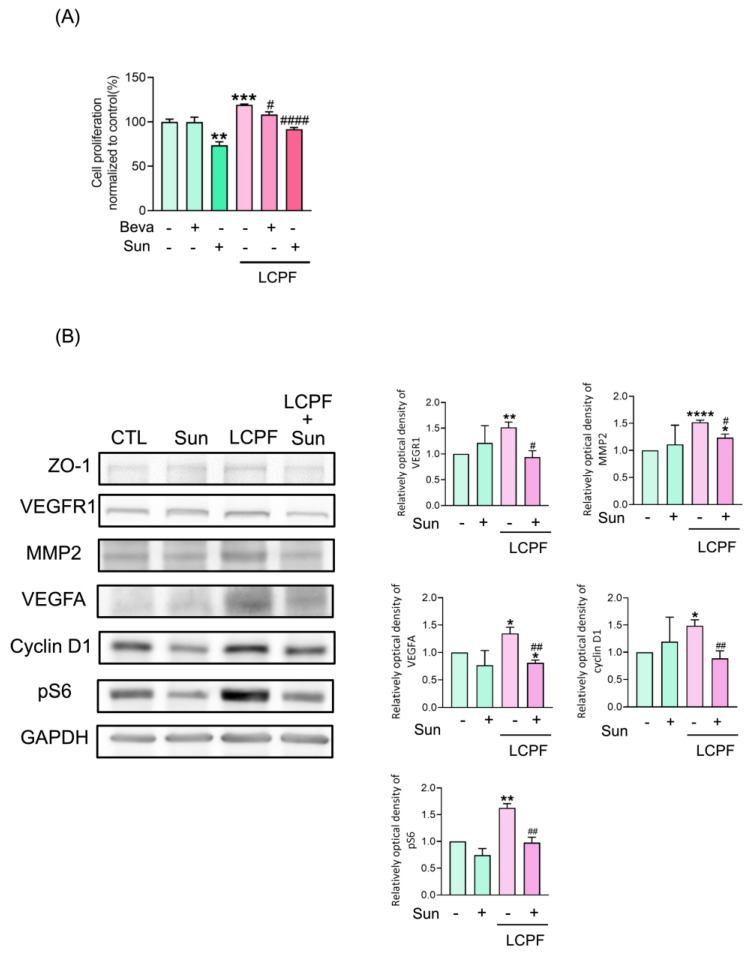
Reverse effect of VEGFR2 inhibitor on keratinocyte cell viability, MMP2, cyclin D1, and pS6 upregulated by LCPF. HaCaT cells were treated with or without 10 µM of sunitinib (Sun), applied as a VEGFR2 inhibitor, in the presence of LCPF for 24 h. (**A**) Cell viability was assessed using an MTT assay. (**B**) MMP2, VEGFA, p21, and pS6 protein expression was examined via Western blot analysis. * *p* < 0.05; ** *p* < 0.01; *** *p* < 0.005; **** *p* < 0.0001 compared to the control group. ^#^ *p* < 0.05; ^##^ *p* < 0.01; ^####^ *p* < 0.0001 compared to the corresponding LCPF group.

**Figure 7:**
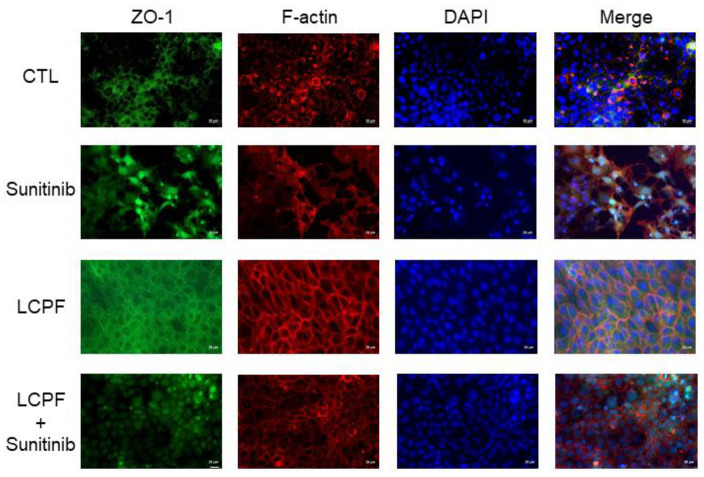
Abrogation of LCPF-induced epidermal junction rearrangement via VEGFR2 inhibitor. HaCaT cells were treated with or without 10 µM of sunitinib (Sun), applied as a VEGFR2 inhibitor, in the presence of LCPF for 24 h. Cells were then subjected to staining for ZO-1 (green), F-actin (red), and DAPI (blue). Scale bar = 40 mm.

When sunitinib was applied as a VEGFR2 inhibitor and bevacizumab as an anti-VEGFA antibody, the MTT assay revealed a significant decrease in HaCaT cell viability after co-treatment with LCPF and sunitinib for 24 h (Figure 6A). Sunitinib treatment attenuated LCPF-induced ZO-1, MMP2, cyclin D1, and pS6 upregulation in HaCaT cells (Figure 6B). The organization of ZO-1 and F-actin is essential for the orchestration of the keratinocyte barrier during skin re-epithelization. We performed immunofluorescence staining of ZO-1 and F-actin to investigate the effect of LCPF and sunitinib on keratinocyte junctional proteins. Compared with that of the control group, HaCaT cells cultured with LCPF showed the increased density of ZO-1 staining in the intercellular space with a reticular appearance (Figure 7). Similarly, F-actin staining of the LCPF group showed a continuous linear distribution at the cell membrane. Accordingly, keratinocytes cultured with LCPF displayed more intact cell junctions, which might benefit skin barrier repair after wounding. In contrast, sunitinib treatment disrupted the effect of LCPF on keratinocyte cell junctions with the loss of ZO-1 and F-actin localization at cell–cell contacts. Additionally, a non-significant change in cytoskeletal assembly was observed when co-treated with LCPF and p-PI3K inhibitors (Figure S3). In summary, VEGFR2 upregulation was involved in LCPF-induced keratinocyte cell viability and junction integrity. 

### 3.4. Application of LCPF-Based Wet Dressing Improves Early Cutaneous Wound Closure in Mice through Increased Re-Epithelization and Collagen Deposition

**Figure 8:**
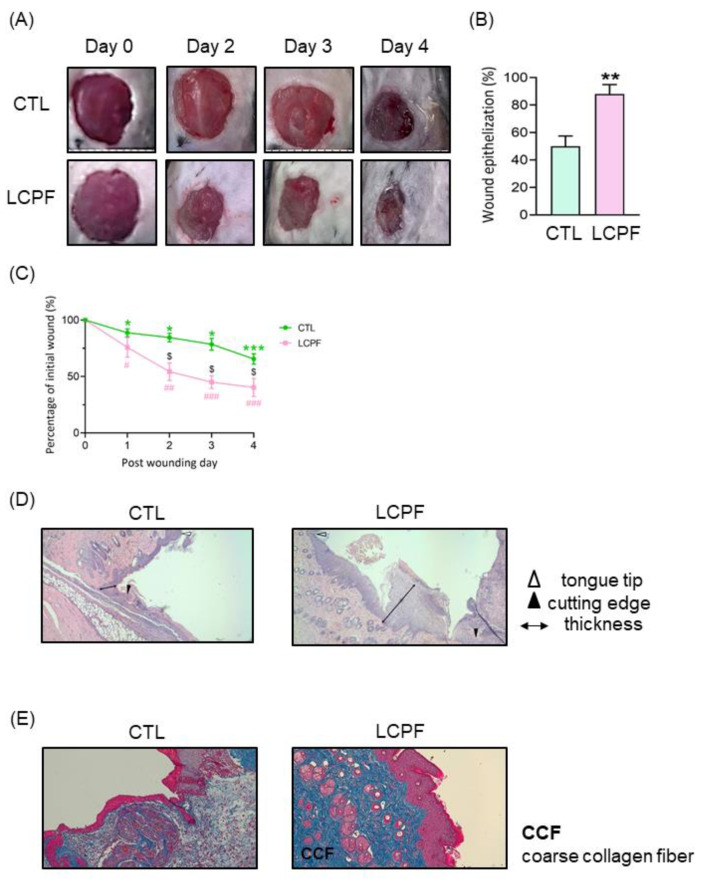
Accelerated wound closure via LCPF-based wet dressing in the mouse skin wound model with increased re-epithelization and collagen synthesis. Using a 6 mm biopsy punch, two full-thickness excision wounds were created on the dorsal skin of the mice. Following the operation, the LCPF-based wet dressing was immediately applied to the left dorsal wound and changed daily. Wet dressing with 0.9% saline served as a control on the right dorsal wound. (**A**) Representative photographs of wounds on different days after wounding. (**B**) The bar graph shows the comparison of epithelization area between the CTL- and LCPF-treated groups. (**C**) The graph shows the time-course changes in the relative percentage of initial wound area between the CTL- and LCPF-treated groups. (**D**) Histological sections of wounds treated with topical 0.9% saline and LCPF at day 4 post-operation (tongue tip; cutting edge; thickness). (**E**) Collagen deposition between wound wet dressing with 0.9% saline and LCPF at day 4 post-operation using Mason trichome staining. (CCF, coarse collagen fiber.) * *p* < 0.05; ** *p* < 0.01; *** *p* <0.005ompared to the initial wound area of the CTL group. ^#^ *p* < 0.05; ^##^ *p* < 0.01; ^###^ *p* < 0.0001 compared to the LCPF group. ^$^ *p* < 0.05 compared to the change in wound area percentage of the CTL group.

To evaluate wound closure via LCPF in vivo, we used two excisional wounds on the dorsal skin of each mouse. The dressing of the wound was changed daily with LCPF or 0.9% saline as a vehicle control. For all experimental groups, wound closure was photographed and analyzed each day. Gross examination revealed that the topical application of LCPF significantly accelerated wound closure kinetics with increased neo-epidermis formation (Figure 8A). Four days after injury, the wound areas recovered to 40% of the original size in the LCPF-based wet dressing group (Figure 8B). Comparatively, the injured areas retained 65% of the original wound in the 0.9%-saline-treated group (Figure 8B). Moreover, the area of neo-epidermis significantly increased to > 80% in the LCPF-treated group (Figure 8C). With regard to cutaneous wound repair, neighboring keratinocytes begin to migrate and were composed of a wedge-shaped epithelial tongue at the wound edge [28]. Compared with macroscopic inspection, histological sections demonstrated increased epithelial tongue lengths and neo-epidermis thicknesses in LCPF-treated wounds on day 4 post-injury (Figure 8D). Some wound studies adopted a splint ring, which was applied to prevent local skin contraction that required 2–4 weeks for wounds to heal [10]. Comparatively, the current study focused on the early stage of wound healing, and the splint was therefore not used. The interference of local skin contraction needed to be considered when evaluating our animal data. In addition, more coarse collagen fibers were noticed in the dermis layer of the LCPF-treated group (Figure 8E). Collagen, as a key component of the extracellular matrix, had a critical role in wound remodeling, and many collagen-based biomaterials have thus been proposed with regard to applications to wound care [29]. Our study demonstrated that the topical use of LCPF could upregulate the collagen thickness in the dermis layer. The above results support the concept that LCPF can be topically applied to accelerate cutaneous wound closure in a full-thickness wound model through re-epithelization and collagen synthesis.

**Figure 9:**
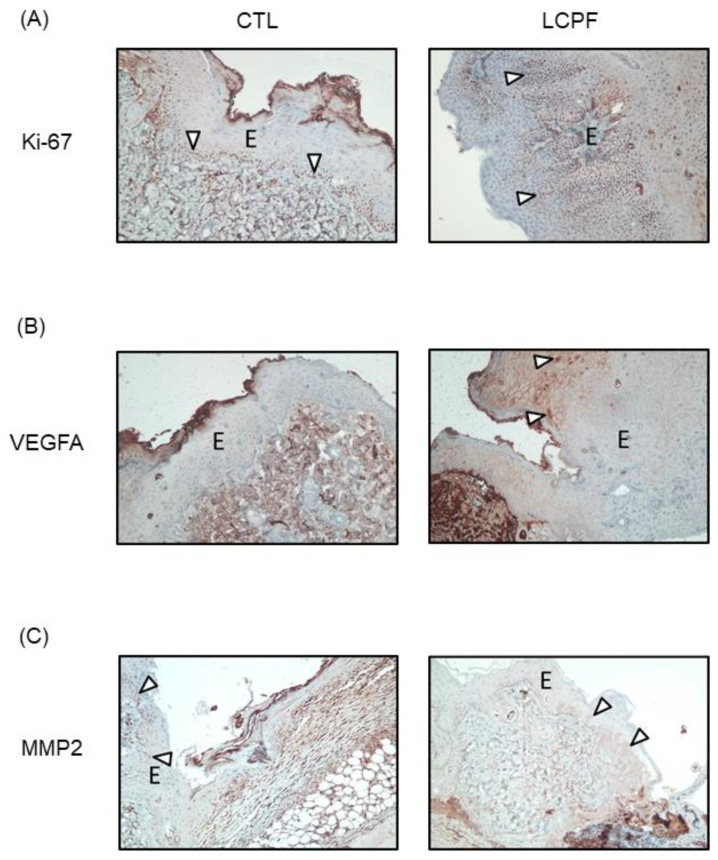
Immunohistochemical analysis of Ki-67, VEGFA, and MMP2 in a full-thickness wound treated with LCPF. Representative images of wounded skin at day 4 post-operation stained with (**A**) Ki-67, (**B**) VEGFA, and (**C**) MMP2. The right panel demonstrates a dorsal wound treated with LCPF wet dressing. The left panel shows a dorsal wound treated with 0.9% saline as the control (positive staining cells; E, epidermis).

Adjacent to the migrating epithelial tongue, an increased thickness of the spinous layer composed of actively proliferative keratinocytes was found [30]. Immunostaining revealed that there were more Ki-67 + proliferating basal cells in the LCPF-treated wounds (Figure 9A). In addition to stimulating angiogenesis, VEGF has been found to directly affect keratinocyte activity during wound healing [31]. LCPF treatment resulted in enhanced staining of VEGFA in the epidermal layer (Figure 9B). MMP2 upregulation plays a critical role in ECM remodeling during wound repair [32]. In the spinous layer adjacent to the epithelial tongue, there was increased MMP2 expression in the LCPF-treated wounds (Figure 9C). Along with microscopic findings of improved skin closure, LCPF treatment upregulated the expression of Ki-67, VEGFA, and MMP2 in the basal layer of the neo-epidermis.

## 4. Discussion

Keratinocyte activity is critical for successful wound healing, and culture with pleural fluid stimulates HaCaT proliferation, migration, and cell cycle progression [1]. Numerous growth factors were involved in cutaneous wound healing, such as VEGF, bFGF, and HGF. Previous research has characterized the presence of multiple growth factors in MPE, including VEGF, bFGF, and PDGF [33,34,35,36]. Ishimoto et al. reported an average VEGF level of 1350 pg/mL in MPE patients [25]. In HFPF, Sack et al. identified a variation in VEGF level of 196 ± 532 pg/mL [26]. We also quantified the level of VEGF, EGF, FGF, and HGF among pleural fluid samples. However, due to the limited sample size, the ELISA data discussed above are summarized in Supplementary Figure S3. The VEGFA levels in LCPF and BCPF were significantly higher. The multiplicity of growth factor might account for the propensity of pleural fluid to increase keratinocyte proliferation and migration. Moreover, we compared the effect of VEGFA and LCPF on keratinocyte migration in Supplementary Figure S4. The group of LCPF showed a superior effect on stimulating HaCaT motility, compared with sole use of VEGF. This might raise the possibility of cell-free pleural fluid as a substitute of growth factor in clinical wound care.

In acute wounding, keratinocytes secrete MMP2 to properly regulate their migration, and thus, re-epithelization occurs [27]. MMP2 upregulation in keratinocytes cultured with pleural fluid further supports the function of pleural fluid in acute skin wounds. Despite the fact that they have the same capacity to induce keratinocyte proliferation, major biological factors related to improving wound closure could differ between HFPF and LCPF. A higher VEGFA level might account for a higher level of keratinocyte proliferation and migration induced by LCPF. Moreover, the effect of stimulating keratinocyte proliferation in HFPF was less dependent on VEGF. More research was required to identify the critical growth factor in HFPF to improve wound closure, such as EGF, FGF, or HGF. Our cellular experiments demonstrated the potential of HFPF and LCPF in accelerating wound closure. PI3K phosphorylation activates mTOR through Akt to promote keratinocyte proliferation and differentiation in acute wounding [28,30]. Additionally, ribosomal protein S6 (pS6) phosphorylation can serve as an indicator of mTOR activity [31]. By applying LY294002 as a PI3K inhibitor, our results demonstrated that LCPF stimulated keratinocyte cell viability via PI3K/Akt signaling and pS6 upregulation. The activation of PI3K/Akt in LCPF-cultured keratinocytes revealed the potential application of pleural fluid in cutaneous wound closure. Targeting VEGFA and VEGFR has shown therapeutic benefits in non-healing wounds [32]. Treatment with sunitinib, a VEGFR2 inhibitor, reversed LCPF-induced keratinocyte cell viability and cytoskeletal rearrangement. Moreover, LCPF-induced MMP2 upregulation in keratinocytes was alleviated. Both VEGFR2 and VEGFA stimulate keratinocyte proliferation at the wound edge [37,38]. VEGFR2 upregulation in keratinocytes was responsible for the therapeutic effect of LCPF on cell viability, migration, and the epithelial barrier.

The topical use of growth factors has been proven to have therapeutic benefits in diabetic wound models, particularly PRP [39,40,41]. The advantages of PRP include its autologous nature and the composition of multiple growth factors [41]. Owing to the abundance of growth factors present in LCPF, we further explored the efficacy of wet dressing with LCPF with better re-epithelization in acute wounding. Regardless of the production of recombinant growth factor or PRP required from patient blood, the final volume required to treat large wound areas remains inefficient in terms of cost [42]. Thoracocentesis often yields >500 mL of pleural fluid; therefore, the application of pleural fluid in wound closure is not limited to the wound surface area. The histological examination of PRP-treated cutaneous wounds revealed typical epithelial regeneration, granulation tissue development, and the neoformation of microvessels [43]. Increased epithelial tongue length and underlying collagen deposition were both observed in wound sections of the LCPF-treated group, suggesting ideal wound closure. PRP was reported to possess different amounts of bioactive substances among individuals [44]. Our preliminary data also showed varied levels of growth factors in HFPF and LCPF (Figure S3). In terms of future research, a mixture of several growth factors might not be powerful enough to mimic the effect of HFPF and LCPF. Additionally, the careful quantification of growth factor content was required following the clinical application of pleural fluid in wound closure. Keratinocytes in the basal layer are mitotically active during cutaneous wound healing [45,46]. Our immunohistochemistry analysis showed increased Ki-67 staining in the basal layer of LCPF-treated wounds, indicating active keratinocyte proliferation. MMP2 expression has been investigated as an indicator of ideal wound healing [47]. The positive staining of MMP2 in LCPF-treated wounds implied the process of active wound closure. Moreover, animal studies have shown that keratinocytes secrete VEGFA in the early stages of wound healing [37]. Our results further demonstrated that the topical use of LCPF induced VEGFA expression in the basal layer of keratinocytes. The above results indicated that wet dressing with LCPF potentially upregulated the basal cell layer of the epidermis with increased Ki-67, MMP2, and VEGFA expression.

## 5. Conclusions

**Figure 10:**
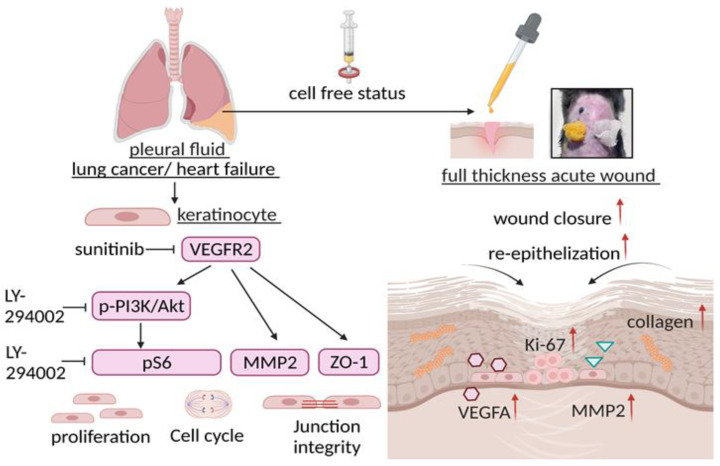
Scheme of LCPF-induced, early cutaneous wound healing and keratinocyte proliferation. Representative images of a mouse cutaneous wound with wet wrap therapy. The left side shows the wet dressing with LCPF and the right shows 0.9% saline as the control.

The current study investigated the potential of cell-free pleural fluid in vitro and in vivo using keratinocytes and a full-thickness skin wound model, respectively (Figure 10). Both LCPF and HFPF promoted keratinocyte proliferation, migration, and cell cycle progression. The upregulation of VEGFR2/VEGFA and PI3K/Akt/pS6 signaling was involved in LCPF-activated keratinocyte proliferation and junction integrity. Wound histology revealed that the topical use of LCPF accelerated re-epithelization and collagen deposition. The similarities between tumor growth and wound healing have been recognized because of the shared features of sustained cell proliferative signaling [48,49]. Our study provides evidence of LCPF as an outside-the-box treatment for cutaneous wound closure. The current study focused on the application of LCPF on early stages of wounds. However, the issue of cancer-related small molecules, including RNAs, or fragments of DNA should be considered owing to mutagenic properties [50]. The possibility of malignant transformation after long term use on cutaneous wounds should be avoided. Advanced techniques to separate carcinogenic RNA or DNA from pleural fluid samples are needed in future clinical applications. 

**Figure 11:**
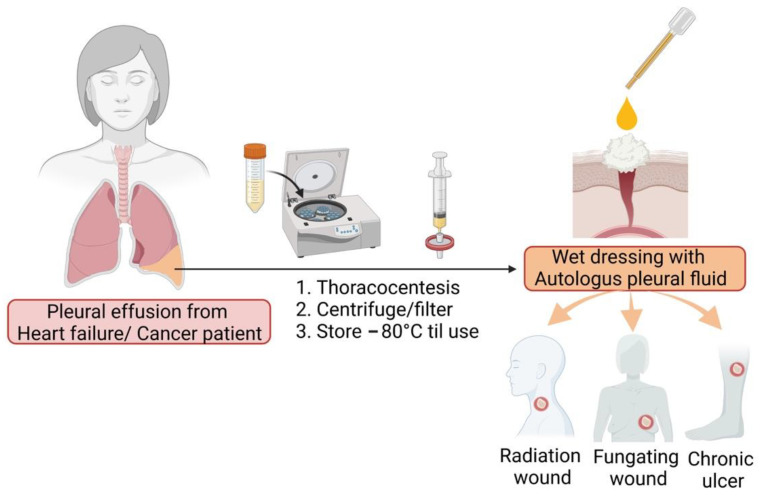
Proposal for the use of autologous pleural fluid in treating cancer patients with non-healing wounds.

Patients with terminal cancer have unmet needs in terms of wound care, such as in the case of fungating wounds in breast cancer, non-healing wounds caused by radiotherapy, and chronic ulcers due to malnutrition. The above conditions significantly impair the quality of life of patients with cancer. Another frequent complication in end-stage patients is malignant pleural effusion (MPE). In this study, cancer-associated pleural fluid was characterized to have an abundance of growth factors. Our preliminary studies showed the wound-healing potential of autologous pleural fluid on cutaneous wound closure with re-epithelization and collagen synthesis. As a result, we proposed the early processing and storage of autologous pleural fluid for later application in wound healing for cancer patients (Figure 11).

## Data Availability

Not applicable.

## Supplementary Materials

The following supporting information can be downloaded at: https://www.mdpi.com/article/10.3390/cells11050817/s1, Figure S1: Effect of PF from breast cancer patients on HaCaT cell proliferation, cell migration, and cell cycle regulation. Figure S2: Effect of p-PI3K inhibitor on HFPF-regulated keratinocyte cell viability. Figure S3: Concentration of VEGF, epidermal growth factor (EGF), fibroblast growth factor (FGF), and hepatocyte growth factor (HGF) in cell free pleural fluid. Figure S4: Comparison of the effect of VEGFA and LCPF on keratinocyte migration.
