# Supplementary material for: Accelerated Wound Healing and Keratinocyte Proliferation through PI3K/Akt/pS6 and VEGFR2 Signaling by Topical Use of Pleural Fluid
Source: Cells. 2022 Feb 26;11(5):817. doi: 10.3390/cells11050817 (PMC8909204; doi:10.3390/cells11050817)
Supplement: Supplementary file 1 [file cells-11-00817-s001.zip › cells-1550348-supplementary.pdf]

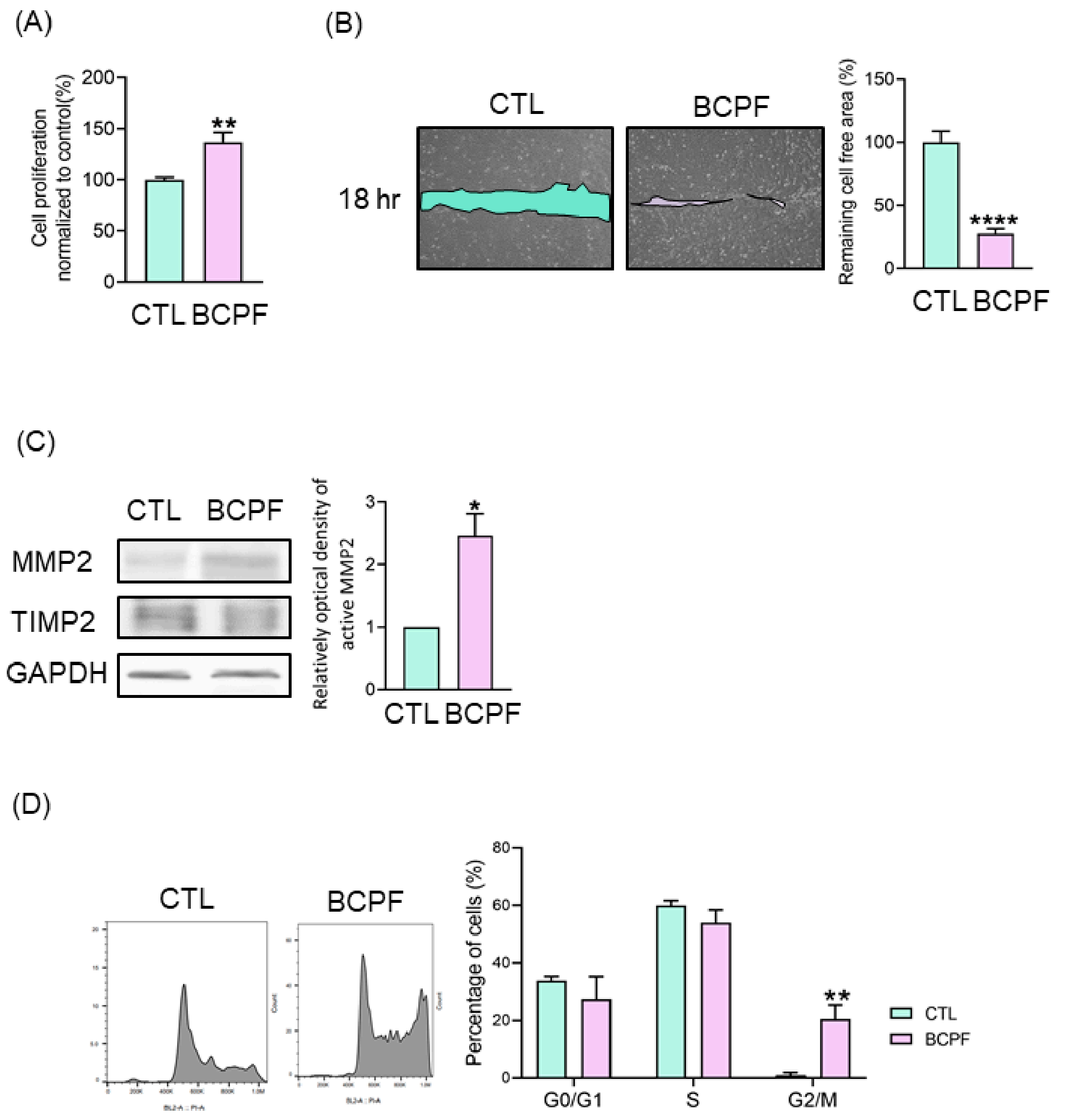

Supplementary Figure S1. Effect of PF from breast cancer patients on HaCaT cell proliferation, cell migration, and cell cycle regulation. Through sonography-guided thoracentesis, PF from breast cancer patients (BCPF) were collected. (A) HaCaT cells were cultured with BCPF or control medium for 24 h. Cell viability was determined using an MTT assay. Quantification shown as bar graphs. (B) After cells reached confluency, a scratch wound assay was applied. HaCaT cells were treated with BCPF or control medium for 18 h. The wound closure is demonstrated by the colored area.

Bar graphs showing the quantification of the cell-free area. (C) Levels of MMP2 and TIMP2 following treatment with BCPF or control medium for 24 h were measured by western blotting. GAPDH was used as an internal control. (D) Flow cytometry was then applied for cell cycle distribution analysis. Quantification of cell populations in the G0/G1, S, and G2/M phases were analyzed using BD FACSuite software. * $p < 0.05$; **$p < 0.01$; ****$p < 0.0001$ compared to the control group. Insignificant changes of tube length and branch point in HUVEC tube formation cultured with BAPF.

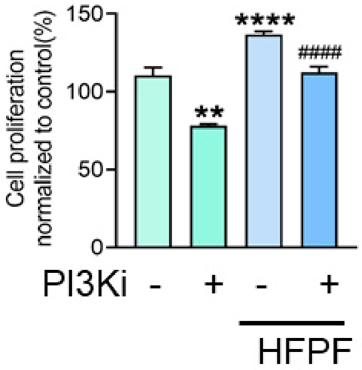

Supplementary Figure S2. Effect of p-PI3K inhibitor on HFPF-regulated keratinocyte cell viability. HaCaT cells were treated with or without 10 μM p-PI3K inhibitor, in the presence of LCPF for 24 h. Cell viability was determined using an MTT assay. **$p < 0.01$; ****$p < 0.0001$ compared to the control group. #### $p < 0.0001$ compared to the LCPF group.

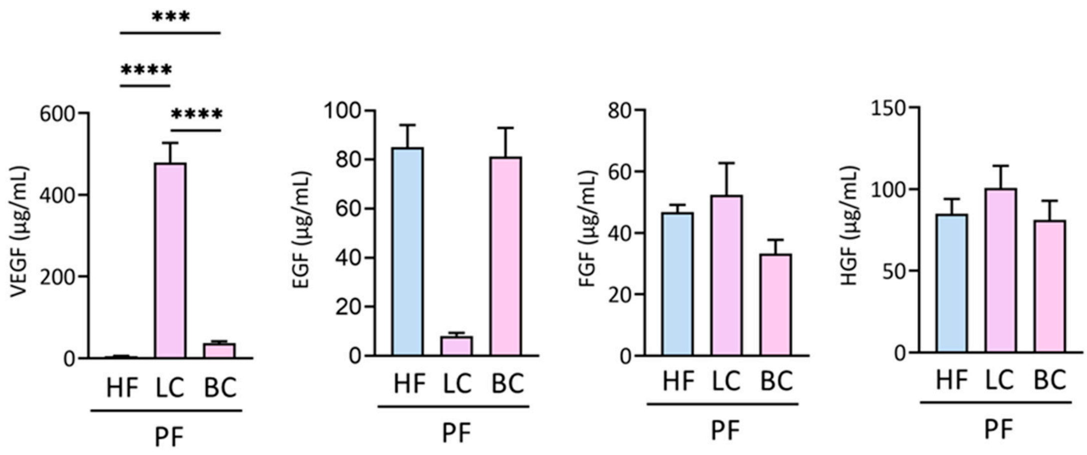

Supplementary Figure S3. Concentration of VEGF, epidermal growth factor (EGF), fibroblast growth factor (FGF), and hepatocyte growth factor (HGF) in cell free pleural fluid. The concentration of growth factors in HFPF, LCPF and BCPF were determined with ELISA kits. *** $p < 0.005$; ****$p < 0.0001$ compared to the other group.

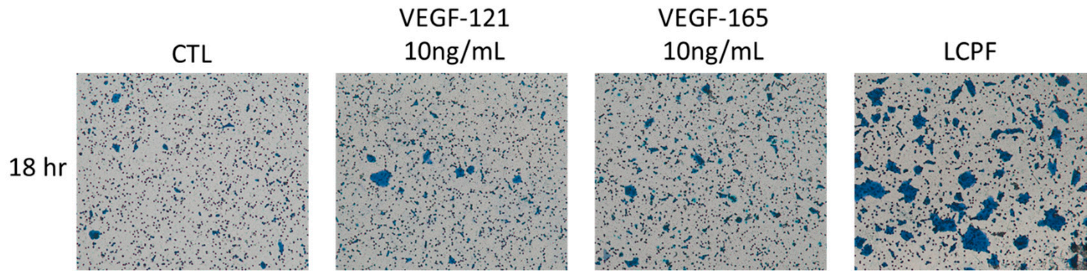

Supplementary Figure S4. Comparison of the effect of VEGFA and LCPF on keratinocyte migration. HaCaT was seeded in the upper chamber of a Transwell plate. After 18 h of culture with VEGFA or LCPF, the cells located in the lower chamber were counted (N = 3). Two VEGFA splice variants, VEGF-121 and VEGF-165 were used with the concentration of 10 ng/mL.
